# A Blockchain Copyright Protection Scheme Based on CP-ABE Scheme with Policy Update

**DOI:** 10.3390/s24144493

**Published:** 2024-07-11

**Authors:** Jian Jiang, Yulong Gao, Yufei Gong, Zhengtao Jiang

**Affiliations:** State Key Laboratory of Media Convergence and Communication, Communication University of China, Beijing 100024, China; jiangjian@cdcgc.com.cn (J.J.); 2021212063037@cuc.edu.cn (Y.G.); z.t.jiang@163.com (Z.J.)

**Keywords:** blockchain, copyright protection, attribute-based encryption, lattice, decision R-LWE

## Abstract

Although the copyright protection schemes supported by blockchain have significantly changed traditional copyright data management, there are still some data security challenges that cannot be ignored, especially the secure access and controllable management of copyright data. Quantum computing attacks also pose a threat to its security. Targeting these issues, we design and propose a blockchain copyright protection scheme based on attribute-based encryption (ABE). In this scheme, the security advantages of blockchain technology are utilized to ensure the authenticity and integrity of copyright data. Based on lattice cryptography and the decision ring learning with errors (R-LWE) problem, a new ABE algorithm that supports searchable ciphertext and policy updates is designed. Then, we introduce it into the blockchain copyright protection scheme, which enables secure access to copyright data and fine-grained control. In addition, the lattice cryptography can strengthen this scheme against quantum attacks. Through security analysis, our scheme can prove to be secure against adaptive chosen keyword attacks, selective chosen plaintext attacks, and adaptive chosen policy attacks in the random oracle model. More importantly, the comparison analysis and experimental results show that our proposed approach has lower computation costs and storage costs. Therefore, our scheme has better security and performance in copyright protection.

## 1. Introduction

Traditional digital copyright protection faces multiple challenges, with frequent piracy and infringement. In the digital era, the reproduction and dissemination of works become extremely easy, leading to the prosperity of the piracy market, causing huge economic losses to copyright owners, and it is still very difficult to trace and maintain copyright on the Internet. At the same time, the vulnerabilities and risks of the centralized platform cannot be ignored. The centralized copyright protection platform is vulnerable to hacker attacks, resulting in copyright information being tampered with or disclosed [1]. In view of the many problems existing in copyright protection, blockchain technology, as an emerging technical means, is widely used in the field of digital copyright protection. Blockchain technology provides a new solution for digital copyright protection through the characteristics of decentralization, tamper proof, and transparency [2]. First, blockchain technology can ensure the uniqueness and tamper resistance of digital content. By storing copyright information on distributed ledgers and using encryption algorithms for verification, the ownership and integrity of works can be effectively protected [3]. Secondly, blockchain can establish smart contracts to manage and protect copyright. Smart contracts can automate the implementation of copyright use rules and trading conditions, reducing the risk of copyright disputes. In addition, blockchain technology can also realize the transparency and traceability of copyright information, providing a fairer and more reliable environment for copyright transactions [4]. In 2019, Zhang et al. proposed a decentralized digital rights management and transaction system architecture based on blockchain technology [5], which is built in a way that consumes fewer resources and throughput.

Although blockchain has shown great potential in digital copyright protection, there are also some new data security challenges, especially data privacy protection [6]. Consensus and storage of copyright data in the blockchain public ledger while ensuring sensitive privacy is not compromised and resisting quantum computing attack is an urgent issue that needs to be addressed [7,8]. Attribute-based encryption (ABE) is an extension of identity-based encryption and is applied in areas such as the Internet of Things and cloud computing [9]. It has encryption advantages and can achieve user privacy and security management in access control. More specifically, in the ABE scheme, the encryptor formulates access policies based on the user’s characteristic information. The user determines whether they can decrypt the ciphertext based on their own characteristics, and the user’s characteristic information is defined as an attribute. Wang et al. proposed a special ABE scheme with revocation support and flexible access structure on the lattice by using a binary tree [10]. Meanwhile, public-key encryption with keyword search (PEKS) enables users to search target encrypted data by keywords, which increases data privacy [11]. In 2019, Liu et al. proposed a KS-ABE scheme to enhance the security of ciphertext search in cloud storage. Meanwhile, this scheme can resist quantum attacks under the LWE assumption [12]. In 2020, Behnia et al. proposed two PEKS schemes using the NTRU lattice and the LWE lattice and implemented these schemes on the Amazon Web Services cloud infrastructure [13]. In 2021, Zuo et al. proposed a blockchain-based ciphertext-policy attribute-based encryption (CP-ABE) scheme for cloud data secure sharing without relying on any trusted third parties [14]. Then, Zhang et al. proposed a new lattice-based ABE algorithm, which is used as an access control method for blockchain data to protect its security [15].

In practical applications, CP-ABE is more suitable for scenarios of distributed storage and update policy, as it allows data owners to define authorized attribute sets as access control policies embedded in ciphertext [16]. Each user can obtain a key corresponding to their attribute set, and the condition for successful decryption is that the attribute set meets the access policy. Therefore, CP-ABE is often combined with blockchain technology to achieve data security [17]. Moreover, the policy updates of decision-makers on user attributes are constantly changing, so policy updates are particularly important in the ABE scheme’s practical application. This approach can ensure the privacy and security of blockchain-based copyright data and update user access policies to ensure data security [18]. Therefore, in this study, we design a blockchain copyright protection scheme based on CP-ABE. In our scheme, the security advantages of blockchain technology are utilized to ensure the authenticity and integrity of copyright data. Based on lattice cryptography and the decision ring learning with errors (R-LWE) problem, we propose a new CP-ABE algorithm that supports searchable ciphertext and policy updates. Then, we introduce it into the blockchain copyright protection scheme. This scheme enables secure access to copyright data and fine-grained control. The security of the proposed scheme can be reduced to the γ−SVP problem and significantly improve users’ privacy and system data security in blockchain-enabled systems and applications.

Then, the main contributions of this paper are summarized as follows:

- We propose a lattice-based CP-ABE scheme to improve anti-quantum security for blockchain copyright protection. The lattice assumption can make our scheme more secure against quantum attacks.
- We construct a new blockchain copyright protection scheme based on the CP-ABE scheme with a policy update, which enables secure access to copyright data and fine-grained control. We provide detailed descriptions of the copyright protection processes. The proposed scheme can significantly protect the copyright and system security.
- We prove our scheme is secure against adaptive chosen keyword attacks, selective chosen plaintext attacks, and adaptive chosen policy attacks in the random oracle model. Furthermore, we compare the proposed scheme with similar literature that shows that it has lower computation costs and storage costs.

The rest of the paper is organized as follows. In Section 2, some definitions and lemmas of the lattice theories are presented. In Section 3, we propose a CP-ABE scheme with policy update. The security proof is presented in Section 4. In Section 5, we give the performance analysis and efficiency comparison of our scheme with other schemes. In Section 6, a blockchain copyright protection scheme based on CP-ABE is designed. The conclusions are provided in Section 7.

## 2. Preliminaries

Some definitions and lemmas of the lattice theories are presented in this section, which are in relation to our scheme.

ℝ,ℤ denote the set of all reals and the set of positive integers, respectively. Let ${\mathbb{R}}^{m}$ be the m-dimensional Euclidean vector space with its usual topology. m∈ℤ,n∈ℤ,m≥n and L denote the Λ lattice; the orthogonal lattice corresponding to Λ is represented by $\Lambda^{⊥}$, vector $x={\left(x_{1},x_{2},\cdots ,x_{n-1},x_{n}\right)}^{T}$ in the space ${\mathbb{R}}^{m}$, and its Euclidean norm $\left\|x\right\|=\sqrt{x_{1}^{2}+x_{2}^{2}+\cdots x_{n-1}^{2}+x_{n}^{2}}$.

**Definition** **1.**  *(Lattice) Given n-linearly independent vectors, lattice L generated by them is the set of vectors.*

(1) $$L(v_{1},v_{2},\cdots ,v_{n})=\left\{\sum_{i=1}^{n}a_{i}v_{i}\left|a_{i}\in \mathbb{Z},i=1,\cdots ,n\right.\right\}$$

$V=[v_{1},v_{2},\cdots ,v_{n}]$ is known as the basis of the lattice *L*. The same lattice can be represented by different lattice bases. Given a prime number *q*, a matrix $A\in {\mathbb{Z}}_{q}^{n\times m}$ defines:

(2) $$\Lambda_{q}(A)=\left\{y\in {\mathbb{Z}}^{m}\left|y=A^{T}x\mathrm{mod}q,x\in {\mathbb{Z}}^{n}\right.\right\},$$

(3) $$\Lambda_{q}{}^{⊥}(A)=\left\{y\in {\mathbb{Z}}^{m}\left|Ay=0\mathrm{mod}q\right.\right\}$$

**Definition** **2.**  *(Lattice SIS problem) Given an integer q, a matrix $A\in {\mathbb{Z}}_{q}^{n\times m}$, and a real constant v>0, find a nonzero vector $x\in {\mathbb{Z}}^{m}$ such that Ax≡0modq and $\left\|x\right\|\leq v$.*

Based on the hardness of the SIS problem, for any polynomial-bounded *m*, *v*, and any prime $q\geq v\cdot \omega \sqrt{n\log n}$, solving SIS on the average is as hard as approximating the shortest independent vector problem (SIVP) in the worst case.

Ring learning with errors (R-LWE) was proposed by Lyubashevsky [19]. It operates on the ring $Z_{q}[x]/(f)$, where *f* is an irreducible polynomial, and *q* is a prime. In most cases, $f=x^{n}+1$, where *n* is a power of 2.

**Definition** **3.**  *(Decision R-LWE problem) For $R=Z_{q}[x]/(x^{n}+1)$, $n=2^{k}$, k≥1, q=1mod2n, $a\in R_{q}$, a and S are uniformly and randomly selected, e∈R is an error vector that follows a discrete Gaussian distribution $\Psi_{a}$. Let b=a⋅s+e, $b\in R_{q}$. The decision R-LWE problem is to distinguish between vector group (**a**,**b**) and vector group uniformly and randomly selected on $R_{q}^{2}$.*

**Definition** **4.**  *(Random oracle model based on decision R-LWE problem) Random oracle O will be sampled with equal probability through two samplers defined as follows:*

Pseudo-random oracle *O*_1_ outputs pseudo-random sampling $(a,b)=(a,a\cdot x+e)\in R_{q}^{2}$, where $a\in R_{q}$ is a uniform random vector, $x\in R_{q}$, the small error term **e** satisfies discrete Gaussian distribution.

True random oracle *O*_2_ outputs uniformly random and mutually independent sample $(a,b)\in R_{q}^{2}$ on the domain $R_{q}^{2}$, which are truly completely random.

The decision R-LWE problem allows adversaries to query the oracle many times and make guesses *O_r_*, r∈{1,2} about the sampler based on the obtained samples. Due to the difficulty of the decision R-LWE problem, the advantages Pr[*r* = 1] − Pr[*r* = 2] of any adversary’s guess *O_r_* on *O* are negligible.

**Lemma** **1.**  *For a lattice L with dimensional m and rank n, $c\in {\mathbb{R}}^{m}$, positive real ε<exp(−4π) and $s\geq \eta_{\varepsilon }(L)$ for random x∈L such that $D_{L,s,c}(x)\leq 1+\varepsilon /(1-\varepsilon )2^{-n}$*.

**Lemma** **2.**  *Let q > 2, a matrix $A\in {\mathbb{Z}}_{q}^{n\times m}$, **B** is the basis of $\Lambda_{q}^{⊥}(A)$, and Gaussian parameter $s\geq ||\tilde{B}||\omega (logm)$. Then, any vector $y\in {\mathbb{Z}}_{q}^{n}$ algorithm SamplePre(**A**, **B**, **y**, s) outputs a vector $e\in Z_{q}^{m}$ from a distribution that is statistically close to $D_{\Lambda_{q}^{⊥}(A),s}(x)$.*

**Lemma** **3.**  *For any prime q=poly(n) and any m≥5nlgq, there is a probabilistic polynomial-time algorithm $TrapGen(1^{n})$ that outputs a matrix $A\in {\mathbb{Z}}_{q}^{n\times m}$ and a full-rank set $S\subset \Lambda^{⊥}(A,q)$. The distribution of A is statistically close to uniform over ${\mathbb{Z}}_{q}^{n\times m}$ and the length $\left\|S\right\|\leq L=m^{1+\varepsilon }\wedge \varepsilon >0$.*

**Lemma** **4.**  *Given a matrix $A\in {\mathbb{Z}}_{q}^{n\times m}$ and an m-dimensional lattice $\Lambda_{q}^{⊥}(A)$, input a basis T of the lattice $\Lambda_{q}^{⊥}(A)$, which has a nonsingular matrix $R=T^{-1}$ and $R\in {\mathbb{Z}}^{m\times m}$, then input Gaussian parameter $s\geq \left\|\tilde{T}\right\|m^{d}\omega (lg^{d+1}(m))$, BasisDel(**A**, **R**, **T**, s) can output a basis **B** of $\Lambda^{⊥}(AR^{-1})$ with overwhelming probability $\left\|\tilde{B}\right\|\leq s\sqrt{m}$.*

## 3. CP-ABE Scheme with Policy Update

### 3.1. Formal Definition

Our ABE scheme consists of six probabilistic polynomial time (PPT) algorithms, such as Setup, Index sharing, KeyGen, Encrypt, Decrypt, and Update, as follows:

(1) **Setup.** The algorithm takes security parameters as input, and the system generates public key *PK* and master key *MK*. Among them, the *MK* is kept by the system.
(2) **Index sharing.** The algorithm mainly includes Index-generation, Trapdoor algorithm, and Test algorithm, which returns a result that stores data and data indexes.
(3) **KeyGen.** The algorithm takes *PK*, *MK*, and user access control policy Plc as input. The system generates secret key *sk* for users according to attribute policy *T*.
(4) **Encrypt.** The algorithm takes public key *PK*, attribute policy *T*, and message *M* as input and outputs ciphertext *C*_1_.
(5) **Decrypt.** The algorithm takes the public key *PK*, secret key *sk*, and ciphertext *C* as inputs. Only if the access control policy Plc matches the user attribute policy *T* does the algorithm output plaintext *M*.
(6) **Update.** Input the main public key, update the access policy, ciphertext, and random trapdoors used in the encryption algorithms, and output new ciphertext *C*_2_.

### 3.2. Our Proposed Scheme

According to the algorithm definition in Section 3.1, the specific process of our proposed CP-ABE scheme with policy update is as follows.

**Setup.**

(1) Set user attribute set ${\mathrm{att}}_{i}\in S,i=1,2,...,N$ and the access policy $Plc_{i}$ corresponding to its attribute ${\mathrm{att}}_{i}\in S$.
(2) Calculate $\sigma_{f}\leftarrow 1.13\sqrt{q/2N}$, select parameters $f,g\leftarrow D_{N,\sigma_{f}}$. Then, calculate the Gram–Schmidt norm as follows.
  (4) $$\left||B_{f,g}||\leftarrow MAX(||(g,-f)||,||(\frac{q\bar{f}}{f*\bar{f}+g*\bar{g}},\frac{q\bar{g}}{f*\bar{f}+g*\bar{g}})||\right)$$

If $|B_{f,g}||\leq 1.13\sqrt{q}$, continue; otherwise, go back to select parameters $f,g\leftarrow D_{N,\sigma_{f}}$ again.

(3) Calculate $\rho_{f},\rho_{g},R_{f},R_{g}$ by using the extended Euclidean algorithm as follows. $\rho_{f}\cdot f=R_{f}\mathrm{mod}(x_{N}+1)$, $\rho_{g}\cdot g=R_{g}\mathrm{mod}(x_{N}+1)$,where $\rho_{f},\rho_{g}\in ℜ$, $R_{f},R_{g}\in Z$. If $\gcd(R_{f},R_{g})\neq 1$ or $\gcd(R_{f},q)\neq 1$, go back to select parameters; otherwise, continue.
(4) Run *extended Euclidean* algorithm to get u,v, and satisfying $u\cdot R_{f}+v\cdot R_{g}=1$ and u,v∈Z. Calculate $F=q\cdot v\cdot \rho_{g}$, $G=q\cdot u\cdot \rho_{f}$, $k=\left\lceil \left(F*\bar{g}+G*\bar{g})/(f*\bar{f}+g*\bar{g}\right)\right\rceil \in {ℜ}_{q}$. Thus, reduce *F* and *G* as follows:F←F−k∗f,G←G−k∗g.
(5) Calculate $h=g*f^{-1}\mathrm{mod}q$, $h\in {ℜ}_{q}$, $B=(\begin{array}{cc} A(g) & -A(f)\\ A(G) & -A(F) \end{array})$, $B\in Z_{q}^{2N\times 2N}$. Output public key PK={h} and master key MK={B}.

**Index sharing.** Index sharing includes the *Index-generation* algorithm, *Trapdoor* algorithm, and *Test* algorithm. During the index-sharing process, the data owner first establishes an index trapdoor to generate a data index and records the data index in the information storage. When data users need to obtain data, they send a request to search the information storage and extract the corresponding data index to obtain the corresponding search results.

(1) **Index-generation.** *N* is a power-of-two integer, and the Trapdoor algorithm corresponds to the encrypted keywords ${k\in \{0,1\}}^{*}$ and $t_{1}=H_{1}(k)$, $H_{1}:{\left\{0,1\right\}}^{*}\to Z_{q}^{N}$. Then, randomly select parameters $r_{1},d_{1},d_{2},\omega_{1}$, where $r_{1},d_{1},d_{2}\leftarrow \{-1,0,1\}$, $\omega_{1}\leftarrow {\left\{0,1\right\}}^{N}$. At last, calculate $I_{1}=r_{1}*h+d_{1}\in {ℜ}_{q}$, $I_{2}=r_{1}*t_{1}+d_{2}+\left\lfloor q/2\right\rfloor \omega_{1}\in {ℜ}_{q}$ and generate index results $S_{k}=\{I_{1},I_{2},H_{1}(\omega_{1},I_{2})\}$.
(2) **Trapdoor.** Run *SamplePre* algorithm $\left(s,T_{k})\leftarrow SamplePre(B.\sigma ,(t_{1},0)\right)$, which satisfies $s+T_{k}*h=t_{1}$. Output corresponding trapdoor $T_{k}$ for keywords *k*.
(3) **Test.** Calculate $y=\left\lfloor 2(I_{2}-I_{1}*T_{k})/q\right\rfloor$; If $H_{1}(y,I_{2})=H_{1}(\omega_{1},I_{2})$, and return *d* = 1; otherwise, return *d* = 0.

**Keygen.** Calculate the hash value of the attribute access control policy $t_{2}\leftarrow H_{3}(Plc_{i})\in Z_{q}^{N}$. Run *SamplePre* algorithm $\left(s_{1},s_{2})\leftarrow SamplerPre(B,\delta ,(t_{2},0)\right)$, which satisfies $s_{1}+s_{2}*h=t_{2}$. In this way, output the secret key *sk* = *s*_2_.

**Encrypt.** Randomly select $r_{2},d_{3},d_{4}\leftarrow \{-1,0,1\},\omega_{2}\leftarrow {\left\{0,1\right\}}^{N}$. Calculate $u=r_{2}*h+d_{3}$, $v=r_{2}*t_{2}+d_{4}+\left\lfloor q/2\right\rfloor \omega_{2}$. Then, calculate $c=m⊕H_{4}(\omega_{2},att_{i},v)$, and return the ciphertext {u,v,c}.

**Decrypt.** Calculate $\chi =v-u\cdot s_{2}$, $\omega_{2}\leftarrow \left\lceil 2\chi /q\right\rceil$.Output the original message $m=c⊕H_{1}(\omega_{2},att_{i},v)$.

**Update.** Define access structure $\hat{W}={\hat{W}}^{+}\cup {\hat{W}}^{-}$, message $m=\{m_{0},m_{1},...,m_{n-1}\}\in {\left\{0,1\right\}}^{n}$, which is expressed as polynomial $m(x)=m_{0}+m_{1}x+...+m_{n-1}x^{n-1}\in R_{q}$; randomly and uniformly sample $\left(B_{i}^{+},B_{i}^{-}\right)$ each attribute $x_{i}\in S$, and $B_{i}^{+},B_{i}^{-}\leftarrow R_{q}^{l\times m}$; select random numbers $s,\beta \leftarrow R_{q}$ and sample $e\leftarrow D_{R,\sigma }$, $e_{A}\leftarrow D_{R_{q}^{m},\sigma }$, calculate $c_{1}=\beta s+e=m\left[q/2\right]$ and $c_{A}=A^{T}s+e_{A}\in R_{q}^{m}$; for each attribute $x_{i}\in S$, calculate separately based on the following conditions.

(1) If $x_{i}\in {\hat{W}}^{+}$, sample $e_{i}\leftarrow D_{R_{q}^{m},\sigma }$ and calculate $c_{i}={\left(B_{i}^{+}\right)}^{T}s+e_{i}\in R_{q}^{m}$;
(2) If $x_{i}\in {\hat{W}}^{-}$, sample $e_{i}\leftarrow D_{R_{q}^{m},\sigma }$ and calculate $c_{i}={\left(B_{i}^{-}\right)}^{T}s+e_{i}\in R_{q}^{m}$;
(3) If $x_{i}\in \hat{W}$, sample $e_{i}{}^{+},e_{i}^{-}\leftarrow D_{R_{q}^{m},\sigma }$, calculate $c_{i}^{+}={\left(B_{i}^{+}\right)}^{T}s+e_{i}^{+}\in R_{q}^{m}$ and $c_{i}^{-}={\left(B_{i}^{-}\right)}^{T}s+e_{i}^{-}\in R_{q}^{m}$;

At last, return the updated ciphertext $C^{\prime }=(\hat{W},c_{A},{\left\{c_{i}\right\}}_{x_{i}\in \hat{W}},{\left(c_{i}^{+},c_{i}^{-}\right)}_{x_{i}\in X\backslash \hat{W}},c_{1})$.

## 4. Security Analysis

### 4.1. Correctness

**Theorem** **1.**  *The proposed CP-ABE scheme satisfies the correctness of keyword indexing.*

**Proof.**  Given the master key MK={B}, public key PK={h}, data index structure $s_{k}=\{I_{1},I_{2},H_{1}(\omega_{1},I_{2})\}$, and keyword trapdoor $T_{k}$, this scheme is the output of the correct dependency test algorithm d=1. In this calculation process, the parameter $r_{1},d_{1},d_{2},T_{k}$ are short vectors, so $r_{1}*s+d_{2}-T_{k}*d_{1}$ in the interval (−q/4,q/4). And

(5) $$\begin{array}{ll} I_{2}-I_{1}*T_{k} & =(r_{1}*t_{1}+d_{2}+\left\lfloor q/2\right\rfloor \omega_{1})-(r_{1}*h+d_{1})*T_{k}\\ & =r_{1}*s+d_{2}+\left\lfloor q/2\right\rfloor \omega_{1}-T_{k}*d_{1} \end{array}$$

According to the Formula (5), we have $\left\lceil 2(I_{2}-I_{1}*T_{k})/q\right\rceil =\omega_{i}$. Thus, it is proven that the keyword index in our ABE scheme satisfies correctness. □

**Theorem** **2.**  *The proposed ABE scheme satisfies the correctness of the policy update.*

**Proof.**  If the access policy formulated by the decision-maker is consistent with the user’s attribute set $x_{i}\in S$, then there is $S\cap W^{+}=W^{+}$, $S\cap W^{-}=\emptyset$. If there is ${\tilde{B}}_{i}\in \{B_{i}^{+},B_{i}^{-}\}$

(6) $$\begin{array}{l} a=w_{A}^{T}(A^{T}s)+w_{A}^{T}e_{A}+\sum_{i\in \left[l\right]}w_{i}^{T}({\tilde{B}}_{i}^{T}s)+\sum_{j\in \left[l\right]}w_{j}^{T}e_{j}\\ \;\;={\left(Aw_{A}\right)}^{T}s+w_{A}^{T}e_{A}+\sum_{i\in \left[l\right]}({\left({\tilde{B}}_{i}w_{i}\right)}^{T}s)+\sum_{j\in \left[l\right]}w_{j}^{T}e_{j}\\ \;\;=\beta s+w_{A}^{T}e_{A}+\sum_{i\in \left[l\right]}w_{i}^{T}e_{i} \end{array}$$

$\mu^{\prime }=c_{1}-a$, so we can have

(7) $$\mu^{\prime }=c_{1}-(\beta s+w_{A}^{T}e_{A}+\sum_{i\in \left[l\right]}w_{i}^{T}e_{i})\approx \mu \left[q/2\right]$$

According to the above Formulas (6) and (7), it is proven that the proposed ABE scheme satisfies the correctness of the policy update. □

### 4.2. ABE Keyword Index Security

**Theorem** **3.**  *The proposed ABE scheme satisfies keyword index security, which satisfies the indistinguishability against adaptive chosen keyword attack (IND-CKA) in the random oracle model.*

**Proof.**  Assume Eve is a polynomial-time malicious adversary, and Charlie is a challenger who wants to solve the hard problem with the query results from Eve. Adversary Eve first specifies the challenge keywords $k_{0}$ and $k_{1}$. Adversary Eve and challenger Charlie conduct the following query response game. □

(1) **Setup.** Challenger Charlie sets the algorithm to generate a public key PK={h} and a master key MK={B}; that is, Challenger Charlie saves the master key and sends the public key to adversary Eve.
(2) **Queries 1.** Adversary Eve queries about hash query, index generation query, and trapdoor query with polynomial time. The specific process is as follows.

***H*_1_-query.** Challenger Charlie initializes two empty lists, *L*_1_ and *L*_2_, to save the query results. Adversary Eve queries the hash function *H*_1_ of the non-target access control policy. Challenger Charlie initializes empty lists and uses them to store the query results of the hash function. If the result is already on the lists *L*_1_ and *L*_2_, Charlie will return the result and give it to Eve; if the result does not appear on the list, Challenger Charlie will randomly select parameters ${r^{\prime }}_{2},{d^{\prime }}_{4},{\omega^{\prime }}_{2}$, where ${r^{\prime }}_{2},{d^{\prime }}_{4}\leftarrow \{-1,0,1\}$, ${\omega^{\prime }}_{2}\leftarrow {\left\{0,1\right\}}^{N}$, and calculate $h^{\prime }=H_{1}({\omega^{\prime }}_{2},at{t^{\prime }}_{i},{r^{\prime }}_{2}*{t^{\prime }}_{2}+{d^{\prime }}_{4}+\left\lfloor q/2\right\rfloor {\omega^{\prime }}_{2})$. Challenger Charlie sends the ${t^{\prime }}_{2}=H_{1}(Plc_{i})$ and $h^{\prime }$ to adversary Eve and respectively stores the new result $\left(k_{i},{t^{\prime }}_{2}\right)$ and $\left({r^{\prime }}_{2},{d^{\prime }}_{4},{\omega^{\prime }}_{2},h^{\prime }\right)$ in list *L*_1_ and list *L*_2_.

**Index generation query.** Adversary Eve queries the Index-generation algorithm for keyword $k_{i}$. Challenger Charlie initializes a new list *L*_3_ to store the index-generated query results. When the generated result already exists in *L*_1_, Challenger Charlie directly gives the result ${s^{\prime }}_{k_{i}}$ to adversary Eve. Otherwise, Challenger Charlie first performs a hash *H*_1_ algorithm to get ${I^{\prime }}_{2}$ and $h^{\prime }$, then randomly selects ${d^{\prime }}_{1}\leftarrow \{-1,0,1\}$ and calculates ${I^{\prime }}_{1}={r^{\prime }}_{1}*h^{\prime }+{d^{\prime }}_{1}$, and finally gives the result ${s^{\prime }}_{k_{i}}$ to adversary Eve and stores $\left(k_{i},{I^{\prime }}_{1},{I^{\prime }}_{2},{t^{\prime }}_{1},h^{\prime },{s^{\prime }}_{k_{i}}\right)$ in *L*_3_.

**Trapdoor query.** Adversary Eve performs a polynomial time inquiry on the Trapdoor algorithm of the attribute set $S^{\prime }$. Assuming that adversary Eve has already inquired about the keyword, challenger Charlie runs the Trapdoor algorithm and generates ${T^{\prime }}_{k}$ using the sampling algorithm $\left(S^{\prime },{T^{\prime }}_{k})\leftarrow SamplerPre(B,\sigma ,({t^{\prime }}_{1},0)\right)$. Afterward, Charlie returns ${T^{\prime }}_{k}$ to adversary Eve.

(3) **Challenge.** Adversary Eve randomly selects keywords *k*_1_ and *k*_2_. Challenger Charlie randomly selects keywords *k*_i_ (i={1,2}). If *i* = 1, challenger Charlie executes the Index-generation algorithm and Trapdoor algorithm to regenerate a new index, which is returned to adversary Eve. If *i* = 2, challenger Charlie returns the searchable ciphertext to adversary Eve.
(4) **Queries 2.** Adversary Eve repeats the Query 1 operations multiple times, sets the hash function *H*_1_-query *h* times, and queries the Trapdoor algorithms for non-target sets.
(5) **Guess.** Based on the query results, adversary Eve gives a guess $i^{\prime }=\{1,2\}$ about *i*. If $i^{\prime }=i$ return 1. Otherwise, return 0. If adversary Eve can give a correct conjecture *i* with an undeniable probability ε>0, challenger Charlie can solve the R-LWE difficulty problem with probability ε/h. As the *h* times of query increases, challenger Charlie needs to solve the R-LWE difficulty problem less frequently. Therefore, given the difficulty of the R-LWE problem, the proposed ABE scheme satisfies the keyword index security.

### 4.3. ABE Ciphertext Security

**Theorem** **4.**  *The proposed ABE scheme satisfies ciphertext security, which satisfies the indistinguishability under selective chosen plaintext attack (IND-sCPA) in the random oracle model.*

**Proof.**  As described in the previous subsection, suppose that Eve is a polynomial-time malicious adversary who can successfully attack the proposed scheme with non-negligible probability ε, and Charlie is a challenger who wants to solve the SVP problem on the NTRU lattice with the query results from Eve. Adversary Eve and challenger Charlie conduct the following query response game. □

(1) **Setup.** Adversary Eve assigns the challenge attribute set $S^{\prime }$. Challenger Charlie sets the algorithm to generate a public key PK={h} and a master key MK={B}; that is, Challenger Charlie saves the master key and sends the public key to adversary Eve.
(2) **Queries 1.** Adversary Eve queries about hash query, private key query, and ciphertext query with polynomial time. The specific process is as follows.

***H*_1_-query.** Challenger Charlie initializes two empty lists, *L*_3_ and *L*_4_, to save the query results. Eve queries the hash function *H*_1_ of the non-target access control policy $Plc_{i}$. Challenger Charlie initializes empty lists and uses them to store the query results of the hash function. If the result is already on list *L*_3_ and list *L*_4_, Charlie returns the result ${t^{\prime }}_{2}$ and gives it to Eve. Otherwise, Charlie randomly selects parameters ${r^{\prime }}_{2},{d^{\prime }}_{4},{\omega^{\prime }}_{2}$, where ${r^{\prime }}_{2},{d^{\prime }}_{4}\leftarrow \{-1,0,1\}$, ${\omega^{\prime }}_{2}\leftarrow {\left\{0,1\right\}}^{N}$, and calculates $h^{\prime }=H_{1}({\omega^{\prime }}_{2},at{t^{\prime }}_{i},{r^{\prime }}_{2}*{t^{\prime }}_{2}+{d^{\prime }}_{4}+\left\lfloor q/2\right\rfloor {\omega^{\prime }}_{2})$. Charlie sends the ${t^{\prime }}_{2}=H_{1}(Plc_{i})$ and $h^{\prime }$ to Eve and respectively stores the new result $\left({t^{\prime }}_{2}\right)$ and $\left({r^{\prime }}_{2},{d^{\prime }}_{4},{\omega^{\prime }}_{2},h^{\prime }\right)$ in list *L*_1_ and list *L*_2_.

**Private key query.** Eve queries the private key related to access control policies $Plc_{i}$ for message *m_i_*. Charlie initializes a new list *L*_3_ to store the query results. If the attribute $S^{\prime }$ satisfies the access control policy $Plc_{i}$, Charlie’s returns continue. Otherwise, Charlie executes the Keygen algorithm and uses $\left({s^{\prime }}_{1},{s^{\prime }}_{2})\leftarrow SamplerPre(B,\delta ,({t^{\prime }}_{2},0)\right)$ to obtain ${s^{\prime }}_{2}$ and return it to Eve.

**Ciphertext query.** Eve performs a polynomial time inquiry on the Trapdoor algorithm of the attribute set $S^{\prime }$. If the attribute $S^{\prime }$ satisfies the access control policy $Plc_{i}$, Charlie’s returns continue. Otherwise, according to the encrypt algorithm, Charlie executes $c_{i}=m_{i}⊕H_{4}({\omega^{\prime }}_{2},at{t^{\prime }}_{i},v^{\prime })$ to obtain ciphertext $\left(u^{\prime },v^{\prime },c_{i}\right)$ and return it to Eve.

(3) **Challenge.** Adversary Eve randomly selects messages *m*_1_ and *m*_2_. Charlie randomly selects messages *m*_i_ (i={1,2}). If *i* = 1, challenger executes the encrypt algorithm and sends the corresponding ciphertext to adversary. If *i* = 2, Charlie randomly selects one ciphertext from the ciphertext set to Eve.
(4) **Queries 2.** Eve repeats the Query 1 operations *n* times and queries the private key query and ciphertext query for non-target attribute sets $S^{\prime }$.
(5) **Guess.** Based on these query results, Eve gives a guess $i^{\prime }=\{1,2\}$ about *i*. If $i^{\prime }=i$, return 1. Otherwise, return 0. If the Adversary can give a correct guess with an undeniable probability ε>0, the challenger can solve the γ−SVP problem with probability ε/n. Therefore, under the difficulty of the γ−SVP problem, the proposed ABE scheme satisfies the keyword index security.

### 4.4. ABE Update Policy Security

**Theorem** **5.**  *Under the assumption of the R-LWE decision, the proposed ABE scheme satisfies the updated policy security, which satisfies the IND-sCPA in the random oracle model.*

**Proof.**  As described in the previous subsection, assuming there is an adversary Eve who wins the IND-sCPA game with an undeniable probability ε>0, the probability of challenger Charlie solving the R-LWE problem is ε/2. Eve and Charlie conduct the following query response game. □

(1) **Setup.** Eve declares a query access structure $W^{*}=W^{+}\cup W^{-}$ and sends it to Charlie. Charlie interacts under the oracle machine after receiving the access structure $W^{*}$, and the oracle machine randomly selects uniform random samples and pseudo-random samples. Thus, Charlie obtains $(A,V_{A})\in R_{q}^{l\times m}R_{q}^{m}$.
(2) **Private key query.** Eve queries the private key related to access control policies $Plc_{i}$ for message *m_i_*. This step is the same as the step shown in the proof of Theorem 4.
(3) **Challenge.** Charlie randomly selects messages $m_{0},m_{1}\in R_{q}$ from those submitted by Eve. And according to the Update algorithm, he calculates $c_{0}=v+\mu_{\beta }\left\lceil q/2\right\rceil$, $c_{A}=V_{A}$. If $x_{i}\in W^{+}$, $c_{i}=V_{i}^{+}$. If $x_{i}\in W^{-}$, $c_{i}=V_{i}^{-}$. If $x_{i}\in x/W^{*}$, $c_{i}^{+}=V_{i}^{+}$ and $c_{i}^{-}=V_{i}^{-}$. Afterward, Charlie sends $CT^{*}=(W^{*},c_{A},{\left\{c_{i}\right\}}_{x_{i}\in W^{+}},c_{1})$ to Eve.
(4) **Guess.** Eve repeats the private key query as many times as before. Then, Eve outputs a guess $\beta^{\prime }$ on β. If $\beta =\beta^{\prime }$, the oracle performs pseudo-random sampling on R-LWE. Otherwise, the oracle implements true random sampling.

The results can be divided into two situations. Assuming that the oracle is an R-LWE sampler, the updatable strategy is an effective challenge to ciphertext, and the distribution of ciphertext is consistent with the distribution of the challenger’s and adversary’s games. It can be inferred that the advantage of the adversary is in making correct guesses; otherwise, if the oracle samples are truly random instances, it proves that the updatable strategy is uniformly distributed, so the adversary can obtain a correct guess with a probability of 1/2. Therefore, according to Definition 3 and Definition 4 in Section 2, the proposed ABE scheme satisfies the updated policy security, which satisfies the IND-sCPA in the random oracle model.

## 5. Efficiency

In this section, we analyze the privacy protection scheme for blockchain data based on ABE. The specific analysis mainly includes efficiency analysis and performance analysis. Efficiency analysis mainly focuses on parameter size analysis and calculation time analysis. Efficiency analysis and performance analysis demonstrate the excellent efficiency and performance of this scheme by comparing it with similar schemes.

The parameter sizes of the proposed scheme are compared with similar schemes, mainly including the sizes of public key, private key, index, trapdoor, and ciphertext. The specific comparison results are shown in Table 1.

**Figure 1:**
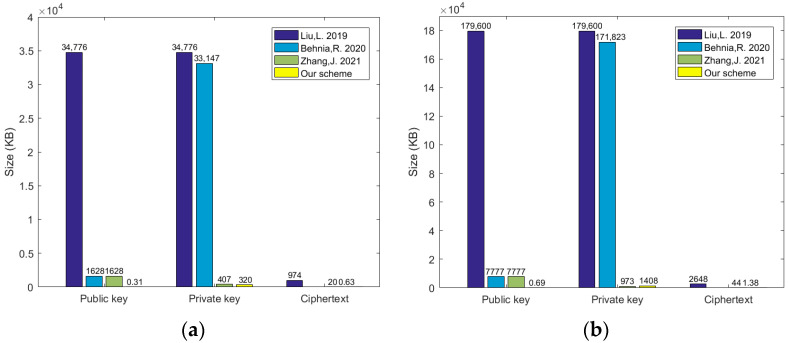
Comparison sizes with other schemes. (**a**) 80-bit security; (**b**) 192-bit security.

Furthermore, the performance and efficiency of these schemes are also analyzed and verified by simulation experiments. The secret key size and ciphertext size of our proposed scheme and those in ABE-PEKS (Liu, L. 2019) [12], LWE-PEKS (Behnia, R. 2020) [13], and Zhang, J. 2021 [15] are compared, respectively. According to the actual requirements in these schemes, under reasonable parameters, we set *n* = 256, *q* = 210, and *m* = 5211 ($m\geq 2n\left\lceil \log q\right\rceil$) with 80-bit security and n = 512, q = 211, m = 11312 ($m\geq 2n\left\lceil \log q\right\rceil$) with 192-bit security. The message length is set to *l* = 64 bit. The public key, private key, and ciphertext sizes of each scheme are compared. The results of the simulation experiments are shown in Figure 1a and Figure 1b, respectively. Here, LWE-PEKS’s ciphertext size is not shown in Figure 1 because the encryption phase does not exist. Through the comparison results, under the same conditions, the public key, private key, and ciphertext sizes in our scheme are significantly reduced, which is conducive to saving the computational overhead in blockchain and optimizing the operation efficiency for our scheme.

In this section, the computational costs of the proposed scheme and similar schemes are analyzed and compared, mainly considering the computational costs of the proposed *Index-generation*, *Trapdoor*, *Test*, *Keygen*, *Encrypt*, and *Decrypt*. As shown in Table 2, *T_h_*, *T_gs_*, *T_sp_*, *T_mul_*, *T_m_*, and *T_e_* are set to represent the average consumption time of the following algorithms: *Hash*, *Gaussain-samplepre*, *Samplepre*, *Modulo-multiplication*, *Matrix operation*, and *Encode operation*, respectively. According to the comparison results in Table 2, it can be concluded that compared with the literature, our proposed scheme has a lower computational time in terms of index generation, encryption algorithm, decryption algorithm, etc. Therefore, our scheme has lower overall computational time costs and higher efficiency.

## 6. Novel Copyright Protection Scheme

### 6.1. Scheme Design

**Figure 2:**
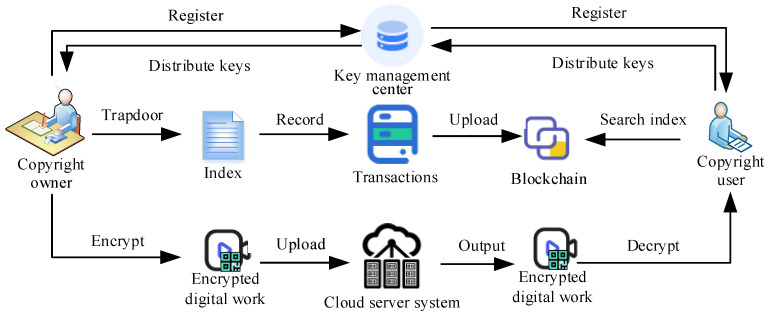
Overview of novel copyright protection scheme.

In this section, a blockchain copyright protection scheme based on the CP-ABE scheme with policy update is designed. As shown in Figure 2, this scheme combines the immutability of blockchain with the flexibility of ABE, aiming to provide a secure and efficient digital copyright protection mechanism. The detailed working process of our new scheme is as follows:

(1) **Initialization.** The blockchain platform serves as the infrastructure for digital copyright protection. There is a secure and reliable key management center in the system, which is responsible for generating and managing the required public key *PK* and master key *MK* for ABE through the *Setup* algorithm and defining the attribute sets and access policies. The attribute set includes various features of digital content, such as author, work name, and release date. The access policy defines which combinations of attributes allow users to access digital content. In addition, through the index sharing algorithm, after the three processes of index generation, trapdoor, and testing mentioned above, a data index structure containing storage addresses and keywords for digital works can be constructed.
(2) **Copyright registration.** Copyright owner registers digital copyright information on the blockchain, including work content, attribute sets, and access policies. By using the *Keygen* algorithm in our ABE scheme, the Key management center generates key *sk* based on registration information and securely distributes them to the corresponding content creators.
(3) **Encryption.** Copyright owner uses attribute-based encryption algorithms to encrypt digital content and embed access policies into ciphertext $c=m⊕H_{4}(\omega_{2},att_{i},v)$. The encrypted digital content is uploaded to the cloud server system, and it is constructed as a transaction record in the blockchain for other users to verify.
(4) **Access control and decryption.** By using the *Index sharing* algorithm, the copyright owner establishes an index trapdoor to generate an index $S_{k}=\{I_{1},I_{2},H_{1}(\omega_{1},I_{2})\}$ that is recorded in the blockchain. Thus, users can find the desired digital work index through keyword indexing and then request the corresponding digital content from the system. Among them, users need to provide their own attribute set proof. Smart contracts on the blockchain are proven based on access policies and user attribute sets to determine whether the user meets the access conditions. If the user meets the access conditions, the smart contract will obtain the corresponding public key from the blockchain and send it to the user. Users use the secret key to decrypt encrypted digital content and obtain the original content.
(5) **Policy Update.** When the copyright owner needs to update the access policy, they execute the *Update* algorithm based on the new access structure, generate new encrypted files, and store them in the system. Smart contracts on the blockchain will automatically update access policies, ensuring that only users who meet the new policies can access digital works.
(6) **Copyright tracing and protection.** If infringement is discovered, the copyright owner can search for registration information and transaction records of digital copyright on the blockchain to prove their copyright ownership. The consensus mechanism of the blockchain ensures the authenticity and integrity of copyright information and provides strong evidence for rights protection.
(7) **Audit and regulation.** Regulatory authorities can regularly audit digital copyright information on the blockchain to ensure the compliance and effectiveness of copyright protection schemes. For discovered violations, regulatory authorities can take corresponding punitive measures to maintain order in the digital copyright market.

Through the above seven stages of work, the blockchain digital copyright protection scheme based on attribute-based encryption with updatable policies can achieve secure, efficient, and flexible digital copyright protection. This not only protects the legitimate rights and interests of creators but also promotes the legitimate dissemination and sharing of digital content.

### 6.2. Advantages of CP-ABE and Blockchain

We design a lightweight blockchain-based copyright protection scheme by recording copyrights on-chain while storing the actual works off-chain. Specifically, the index structure, which contains the storage address and keywords of digital copyright data, is uploaded to the on-chain ledger. Meanwhile, the digital content data, which occupies a substantial storage space, is stored on local servers or cloud servers of various digital media. This approach, combined with CP-ABE and searchable ciphertext, achieves efficient and secure solutions. The specific advantages are as follows:

(1) Unlike traditional copyright protection systems, in our scheme, the on-chain ledger records only lightweight information, such as data storage, verification, operation, and transaction records. Upon generation of the original copyright data, it forms an index structure that is associated with real data, encompassing keywords, storage addresses, and signatures of digital content. This approach enables the proposed scheme to reduce the cost of storage space and enhance the efficiency of blockchain copyright data recording. In addition, the data storage method of blockchain enhances the copyright data’s traceability and integrity.
(2) After encrypting data using the proposed CP-ABE scheme with a searchable encryption algorithm, a trapdoor containing copyright data keywords is generated. Subsequently, the index is recorded as a transaction in the blockchain ledger. Users can search for the desired digital works by embedding keywords in the data index structure beforehand. Upon passing the preset trapdoor verification, access to the data is granted, and the actual digital works can be downloaded from the cloud server through the address information in the index. Additionally, the keyword search functionality of this scheme is highly suitable for cross-institutional sharing of copyright data. On public cloud platforms, this indexing method can establish an effective channel to find the required digital work data through keywords. Furthermore, this approach prevents damage caused by direct human contact with data, thereby effectively protecting the security of digital works.
(3) Due to the large size of digital work files, copyright protection systems have storage requirements for a large amount of audio and video data. However, placing this massive amount of data on the blockchain is impractical. Therefore, utilizing local servers and cloud storage technology, as described in this article, is a more promising and resource-efficient way to address this data storage challenge.
(4) For copyright data, it is first encrypted by CP-ABE, and the encrypted data are stored on the blockchain. Incorporating the attributes of data into the secret key enables users to control their respective copyright data in a more granular manner. Only by verifying the correct attribute access policy can permission to view real data be obtained, thereby ensuring copyright data security.

### 6.3. Scheme Security Analysis

For the copyright protection scheme’s security, firstly, it is necessary to target complex user access and achieve fine-grained management of data access. Secondly, it is extremely important to ensure the copyright data’s security, including copyright data encryption security and privacy security. In addition, the data stored within the system cannot be maliciously tampered with, and the copyright protection system resists single points of failure and data silos. Based on the above security requirements, in this subsection, we conduct a detailed security analysis of the proposed copyright protection scheme.

#### 6.3.1. Fine-Grained Access Control

Our CP-ABE scheme focuses on access control for attribute encryption in copyright protection. The CP-ABE scheme allows for the association of access control policies with encrypted ciphertext data and uses user attributes to define access rules. Only users who meet these attribute conditions can decrypt and access copyright data and conduct copyright transactions. Meanwhile, through the previous security analysis of the CP-ABE scheme, it is proven that the CP-ABE scheme satisfies the correctness of encryption. This CP-ABE technology provides fine-grained access control capabilities while protecting data privacy and ensuring data security. Therefore, our scheme satisfies the security requirements for copyright data’s fine-grained management in copyright protection systems.

#### 6.3.2. Data Security and Privacy Protection

On the one hand, in this paper, CP-ABE is applied to copyright data so that user who satisfies the attributes can decrypt the data, ensuring the privacy and security of the data. Only when the user’s attribute satisfies the access policy can the data be decrypted and obtained. Otherwise, there is no other way to obtain copyright information or complete copyright transactions. The correctness and ciphertext security analysis of the attribute encryption scheme proven in Section 4 proves that the scheme can ensure the privacy and security of copyright data. On the other hand, the proposed CP-ABE scheme is based on lattice. Lattice-based cryptography is generally considered to have the advantage of resisting quantum computing attacks, and it can deal with the threat of quantum computers in the future [20]. Researchers have advocated for the development of novel strategies to include data encryption in the post-quantum era. Subsequently, lattice-based cryptography has garnered significant attention and focus in recent years, with its applications expanding rapidly across multiple domains [21,22]. Therefore, by using the theory of random lattice and the corresponding lattice basis, in this paper, we propose a new lattice-based CP-ABE scheme to improve anti-quantum security for blockchain copyright protection, which can protect data security in the future.

#### 6.3.3. Resist Tampering Attack

If an adversary intends to tamper with existing copyright data and transaction information on the blockchain through tampering attacks, the blockchain can effectively resist tampering attacks through timestamp proof, distributed ledger, digital signature, SHA-256 algorithm, consensus mechanism, etc. Only if the consensus is successful can this transaction be verified and submitted to the blockchain distributed ledger. Therefore, by introducing blockchain technology into this copyright protection scheme, it can effectively prevent individual malicious nodes from tampering with transaction data.

#### 6.3.4. Resist Single Point of Failure and Data Island

By harnessing the power of blockchain technology for decentralized distributed storage and robust authentication of copyright data, this innovative copyright protection scheme addresses the critical issues of single-point failure and data isolation that plague traditional, centralized copyright protection systems, thereby ensuring a more secure and reliable approach. This scheme utilizes the distributed ledger feature of blockchain technology to back up copyright data in a distributed manner on multiple nodes. In this way, even if some nodes fail or are attacked, other nodes can still work normally, ensuring the safe operation of the system and copyright data’s integrity. At the same time, the advantages of secure sharing of blockchain data can improve data island problems and achieve interconnectivity of copyright data. Therefore, by using blockchain for decentralized distributed storage and secure authentication of copyright data, this new copyright protection scheme can effectively solve the single point of failure and data island problems faced by traditional centralized copyright protection systems. In addition, the transparency and traceability of blockchain enhance the transparency of copyright usage and the verifiability of historical change records, providing strong evidence support for copyright protection.

## 7. Conclusions

The application of blockchain in fields such as the Internet of Things and digital copyright has become a trend. This study focuses on the urgent challenges of data privacy security and fine-grained access control that need to be addressed. We have studied and designed an innovative blockchain copyright protection scheme based on CP-ABE, which effectively addresses the data security and privacy challenges in traditional copyright data management, particularly in defense against quantum computing attacks. By utilizing the security and lattice cryptography of blockchain technology, this scheme not only ensures the authenticity and integrity of copyright data but also achieves secure access and fine-grained control of copyright data. In addition, the use of lattice-based cryptography can significantly enhance the scheme’s ability to resist quantum attacks. After security analysis and experimental verification, this scheme demonstrates resistance to multiple attacks under the random oracle model while having lower computational and storage costs, providing an efficient and secure new method for copyright protection.

## Figures and Tables

**Table 1:** Comparison with other schemes based on lattice.

Scheme	ABE-PEKS [12]	LWE-PEKS [13]	Ref. [10]	Ref. [15]	Our Scheme
Public key	(*m*^2^ + *mn* + *n*) log *q*	*mn*log *q*	(5*mn* + 2*n*)log *q*	*mn*log *q*	*n*log *q*
Private key	(*m*^2^ + *mn* + *n*) log *q*	*m*^2^log *q*	2*nlm* log *q*	(*n + lm*)log *q*	4*n*^2^ log *q*
Index	3*mn* log *q*	(2*mn + n*)log *q*	—	—	2*n* log *q*
Trapdoor	2*mn* log *q*	3*mn*log *q*	—	—	2*n*^2^ log q
Ciphertext	(2*n*^2^ + 2*lm* + 1) log *q*	—	(3*nml* + 2)log *q*	(*nl* + 1)log *q*	(2*n* + 1) log *q*

**Table 2:** Time cost comparison with other schemes.

Scheme	ABE-PEKS	LWE-PEKS	Our Scheme
Index-generation	$T_{h}+2T_{mul}+T_{m}$	$T_{h}+2T_{mul}+2T_{m}$	$2T_{h}+2T_{mul}$
Trapdoor	$tT_{h}+2T_{mul}+T_{m}$	$T_{gs}$	$T_{sp}$
Test	$kT_{mul}+T_{gs}$	$T_{mul}$	$T_{h}+T_{mul}$
Keygen	-	$2T_{gs}$	$T_{h}+T_{sp}$
Encrypt	-	$2T_{mul}+2T_{m}$	$T_{h}+2T_{mul}$
Decrypt	-	$2T_{mul}+2T_{m}$	$T_{h}+T_{mul}$

## Data Availability

Data are contained within the article.
